# Growth of a Tessellation: Geometric rules for the Development of Stingray Skeletal Patterns

**DOI:** 10.1002/advs.202407641

**Published:** 2024-11-07

**Authors:** Binru Yang, David Knötel, Jana Ciecierska‐Holmes, Jan Wölfer, Júlia Chaumel, Paul Zaslansky, Daniel Baum, Peter Fratzl, Mason N. Dean

**Affiliations:** ^1^ Max Planck Institute of Colloids and Interfaces Potsdam Science Park Am Mühlenberg 1 OT Golm 14476 Potsdam Germany; ^2^ Cluster of Excellence Matters of Activity Image Space Material Humboldt‐Universität zu Berlin Unter den Linden 6 10099 Berlin Germany; ^3^ Zuse Institute Berlin (ZIB) Takustr. 7 14195 Berlin Germany; ^4^ Institut für Biologie Humboldt‐Universität zu Berlin Philippstraße 13, Haus 2 10115 Berlin Germany; ^5^ Department of Organismic and Evolutionary Biology Harvard University 26 Oxford Street Cambridge MA 02138 USA; ^6^ Department for Operative Preventive and Pediatric Dentistry Charité‐Universitätsmedizin Berlin Aßmannshauser Str. 4–6 14197 Berlin Germany; ^7^ Department of Infectious Diseases & Public Health City University of Hong Kong Hong Kong China; ^8^ Centre for Nature Inspired Engineering City University of Hong Kong Hong Kong China

**Keywords:** bio‐inspired design, biomineralization, cartilage skeleton, mechanobiology, tesserae

## Abstract

The skeletons of sharks and rays, fashioned from cartilage, and armored by a veneer of mineralized tiles (tesserae) present a mathematical challenge: How can the continuous covering be maintained as the skeleton expands? This study, using microCT and custom visual data analyses of growing stingray skeletons, systematically examines tessellation patterns and morphologies of the many thousand interacting tesserae covering the hyomandibula (a skeletal element critical to feeding), over a two‐fold developmental change in hyomandibula length. The number of tesserae remains surprisingly constant, even as the hyomandibula expands isometrically, with all hyomandibulae displaying self‐similar distributions of tesserae shapes/sizes. Although the distribution of tesserae geometries largely agrees with the rules for polyhedra tiling of complex surfaces—dominated by hexagons and a minor fraction of pentagons and heptagons, but very few other polygons—the agreement with Euler's classic mathematical laws is not perfect. Contrary to the assumed uniform growth rate (which is shown would create geometric incompatibilities), larger tesserae grow faster to accommodate skeletal expansion. It is hypothesized that this local regulation of global system complexity is driven by tension (from cartilaginous core expansion) in the fibers connecting tesserae, with strain‐responsive cells orchestrating local mineral apposition.

## Graphical abstract

Mineralized tiles (tesserae) armor stingray skeletal cartilage, somehow growing throughout life without disrupting the tessellation. It is discovered that tesserae size distributions remain strikingly self‐similar across ontogeny. This implies growth isn't uniform, but rather tile geometry dictates mineral apposition in growing tesserae. This can be governed by sensation of tensions in organic fibers connecting neighboring tiles, as the cartilage core expands.

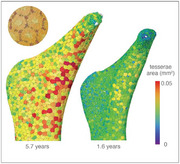

## 1 Introduction

Tessellations are common motifs in nature, where organisms employ arrays of hard tiles on parts of their bodies for protection, stiffening, or other diverse functions, ranging from optics to water transport.^[^ ^1^ ^]^ The skeletons of cartilaginous fishes, such as sharks and rays, for example, are defined by a distinctly tessellated architecture, where mineralized tiles called tesserae cover an otherwise non‐mineralized cartilage skeleton.^[^ ^2^ ^]^ This tessellation has the mechanical function of protecting the comparatively soft cartilaginous elements and providing them with sufficient rigidity to function as a skeleton.^[^ ^2^, ^3^ ^]^

Tessellations present a number of functional advantages over a continuous rigid layer, not least by their resistance to cracking through providing rigidity but also some minimal flexibility.^[^ ^4^ ^]^ These mechanical advantages are clearly leveraged in stiff manmade surface tessellations, from road pavements to floor tilings. However, compared to these architectural elements, the tessellations of shark and ray skeletons need to support the growth of the animal without disrupting the mechanical integrity of the skeleton. This is an impressive feat, as sharks and rays continue to grow throughout their lifetimes, which can span from decades to hundreds of years,^[^ ^5^ ^]^ and involve a several‐fold increase in body size.^[^ ^6^ ^]^ Additionally, whereas the multiple cell types in mammalian bone allow tissue deposition, resorption, and remodeling,^[^ ^7^ ^]^ the apparent absence of resorption and remodeling processes for shark and ray tessellations raises the fundamental question of how they can support continuous skeletal growth, while maintaining an integral surface covering.

In principle, skeletal growth could either be supported by an increase in the number of tesserae, by the growth of individual tesserae, or both. Indeed, tesserae have been shown to be capable of increasing in size during growth: tesserae are connected to each other laterally by organic fibers that are closely associated with cells. Both the fibers and cells are presumably involved in the deposition of mineralized tissue at the perimeter of tesserae, allowing them to grow.^[^ ^2^, ^8^ ^]^ However, as we shall demonstrate in this study, a constant rate of apposition (i.e., deposition of new mineralized material) at the edges of tesserae would not necessarily result in the growth of a continuous (i.e., unbroken) tessellation. On the contrary, this may even lead to geometric incompatibilities, suggesting that more nuanced growth patterns must be involved in tessellated cartilage.

**Figure 1:**
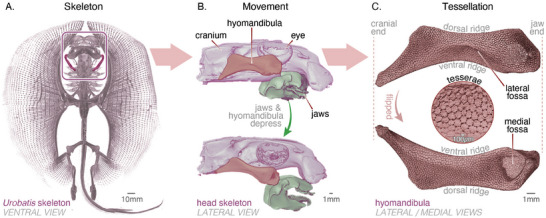
Skeletal anatomy of the stingray *Urobatis halleri*. A) Although comprising mostly cartilage, the skeleton of sharks and rays is visible in micro‐computed tomography (*µ*CT) scans (all images) due to surface mineralization of the cartilage. B) The *hyomandibula* (also highlighted in A) is a skeletal element connecting the cranium and jaw, loaded in bending and twisting when the jaw is protruded (bottom image). C) *µ*CT of the *hyomandibula* reveals the tessellation of the surface covering, comprised of thousands of mineralized tiles called tesserae. Individual tesserae are typically small (≤500 µm in diameter), polygonal and numerous (circular inset). Note that the two anatomical ridges and fossae tend to be associated with distinct tesseral features in our data.

To understand how a natural system solves the architectural conundrum of growing a tessellated surface, we study the tessellation of the *hyomandibula* of Haller's stingray (*Urobatis halleri*), applying advanced segmentation approaches to laboratory micro‐computed tomography (*µ*CT) data as a function of animal age. The *hyomandibula* is an element in the hyoid skeleton, distinguished by broad, and relatively flat surfaces (i.e., with only shallow saddles and depressions; see **Figure** **1**), which facilitates analysis of the tessellated surface, but is also a relatively small skeletal element (≈1.3–2.6 cm long in our dataset). This allows investigation of intact skeletal pieces across a nearly twofold difference in animal body size, while maintaining a high spatial resolution in the *µ*CT scans. Even the smallest *hyomandibula* investigated bore thousands of tesserae which, at the resolution of our *µ*CT datasets, appeared as irregular geometric tiles on the surface of the skeleton, abutting their neighbors (Figure 1). The tesserae covered the majority of the *hyomandibula*, with the exception of a concave medial fossa close to the distal end of the element (Figure 1). Based on the *µ*CT‐data, we analyze how hyomandibular size and shape, as well as the number and characteristics of tesserae change as the animal grows. We show that the distributions of most morphometric parameters evolve in a self‐similar way and that the number of tesserae stays essentially constant across age, although the hyomandibular surface that they cover expands more than 4‐fold during the growth of the animal. We demonstrate theoretically that the observed skeletal growth cannot occur by the accretion of mineralized tissues at a constant rate along tesseral margins and argue that a level of biological control is required to maintain the integrity of the tessellation. Finally, we propose a simple conceptual model to explain how this type of tessellated surface growth might occur, pointing to specific tissue regions essential for coordinating this complex multi‐factor growth in three dimensions.

## 2 The Growing Tessellated Skeleton

**Figure 2:**
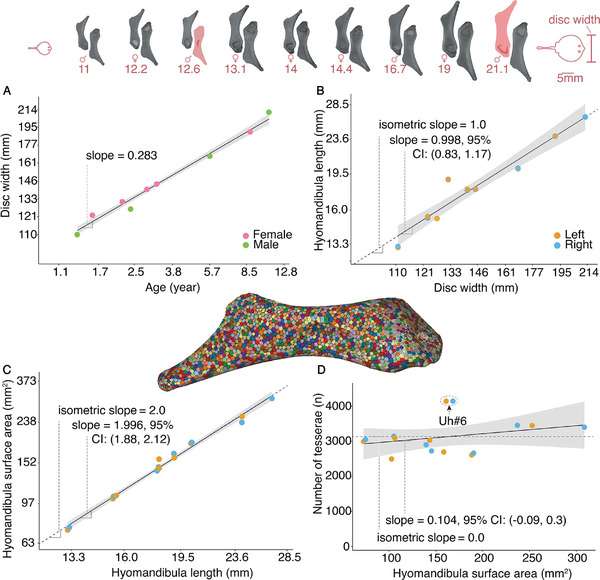
Growth of the *hyomandibula*. A) Disc width is a linear measurement of body dimension used to describe animal size in batoid fishes (rays, skates, guitarfishes^[^ ^10^ ^]^). The individual stingrays we studied are estimated to range from 1.3 to 12.8 years in age (based on existing equations for converting between disc width and age,^[^ ^10^ ^]^ B) with *hyomandibulae* roughly doubling in length across these specimens; *µ*CT scan images of individual animal *hyomandibulae* along the top of the figure are scaled similarly to illustrate this (the two red *hyomandibulae* indicate specimens missing from our dataset). The positive correlations of animal disc width, *hyomandibula* length, and C) *hyomandibula* surface area illustrate that surface area can be used as a proxy for age. The colored volume rendering in the center of the figure illustrates the large number of tesserae per *hyomandibula*. D) Surprisingly, however, the number of tesserae remains nearly constant with age, with a slight increase in the total number of tesserae (10%), indicating that skeletal growth is accomplished mainly by existing tesserae increasing in size. Exceptionally, in one specimen (Uh#6), the number of tesserae is considerably higher, a function of its particularly small tesserae (see Figure 4; Figure S1B, Supporting Information). This deviation was observed in both left and right hyomandibulae, indicating it to be characteristic of this individual, perhaps somehow related to the size of its *hyomandibulae*, which are particularly long for its body size. Note that the horizontal and vertical axes in (A–C) are on logarithmic scales.

*Hyomandibulae* from stingrays of different ages were imaged and analyzed by *µ*CT. The close packing of tesserae on the *hyomandibula* challenges traditional segmentation workflows used to digitally separate objects in reconstructed tomographic data. In standard protocols, image segmentation relies on identifying consistent grayscale differences between foreground and background (i.e., between tesserae and unmineralized tissue), and on the presence of gaps between objects to be isolated (i.e., between tesserae). We therefore adopted a digital segmentation workflow we developed and optimized for tessellated architectures,^[^ ^9^ ^]^ which relies on both grayscale and shape information to isolate tesserae as individual, quantifiable objects. This approach enabled us to digitally quantify the complete tessellation from each *hyomandibula* (see **Figure** **2** skeletal rendering), allowing quantification and comparisons of entire populations of many thousands of tesserae across ontogeny.

Based on published curves correlating body size with age for the stingray species studied,^[^ ^10^ ^]^ our sampled specimens were estimated to range from ≈1 to 13 years of age (Figure 2A; Figure S1A, Supporting Information). As expected, *hyomandibulae* were larger in larger stingrays, increasing in both length and surface area (i.e., the surface available for tessellation) in a nearly perfectly isometric relationship with body size (Figure 2B,C). This shows that the *hyomandibula* does not drastically change its proportions with age, making it suitable as a model for our investigations of tessellated system growth. More importantly, these observations illustrate that *hyomandibula* surface area can be used as an effective proxy for stingray body size and age, which is particularly valuable for our growth studies. The surface area is the most relevant variable for describing the available “substrate” on which tesserae can grow.

Although the *hyomandibula* surface increased by about a factor of four across the specimens studied, tesserae number increased only marginally, with roughly 3000 tesserae covering each *hyomandibula* (Figure 2D). Additionally, the proportion of mineralized tissue (i.e., tesserae) to total hyomandibula volume remained roughly constant across ontogeny (≈17% mineralized tissue by volume; Figure S8, Supporting Information), indicating that the volume ratio of uncalcified and calcified cartilage remains largely constant. The slight positive slope of tesseral number versus surface area suggests some new tesserae may arise during ontogeny, but this strategy and, on the other hand, the fusion of tesserae, are likely to be of minimal importance for growing the skeleton. Instead, given the relatively consistent numbers of tesserae observed across animals in our dataset, enlargement of the many thousands of individual tesserae covering the skeleton must account for the changes we observed in *hyomandibula* length and surface area. Previous data have, indeed, argued this as a growth strategy: in histology and electron microscopy, tesserae exhibit concentric bands of varying mineral density, suggesting accretive growth with no remodeling,^[^ ^8^, ^11^ ^]^ and investigations of small subsamples of several tesserae showed tesserae do tend to be larger in larger animals.^[^ ^8^, ^12^ ^]^ Our data, however, offers the first illustration of these changes occurring at the level of an entire tesserae “population”, including all tesserae from a complete skeletal element. These findings assert that stingray skeletal growth is accomplished by many thousands of mineralization fronts, active simultaneously. This strategy has a multiplicative effect at the skeletal level: a simple estimate shows that at the earliest age, there is a mean accretion rate of ≈45 microns per year at the joint surface of the tesserae, while the growth rate of the whole *hyomandibula* length is ≈6 mm per year. Both rates decrease strongly with animal age (see Figure S9, Supporting Information), in line with the deceleration of stingray growth rates across ontogeny.^[^ ^10^ ^]^

Although sharks and rays can live as long or even far longer than humans,^[^ ^13^ ^]^ our demonstration of the near constancy of tesseral number during stingray skeletal growth indicates that most tesserae in the adult skeleton are established extremely early in skeletal development, defining a pattern that is then maintained throughout life. The factors coordinating where and how many tesserae first appear at the start of skeletal mineralization are unknown, yet the predetermination of tesseral number echoes recent observations of ontogenetic patterning in the armor of boxfish^[^ ^9b^ ^]^ (*Lactoria cornuta*), where the number of scutes in the outer carapace remains constant, despite nearly an order of magnitude increase in fish body size. There are, however, ≈10x more tesserae on the stingray *hyomandibula* than scutes on an entire boxfish carapace, suggesting growth of the tiled skeletal system in tessellated cartilage may be more complicated or at least involve the coordination of far more independently growing elements.

## 3 The Tessellation Toolkit

**Figure 3:**
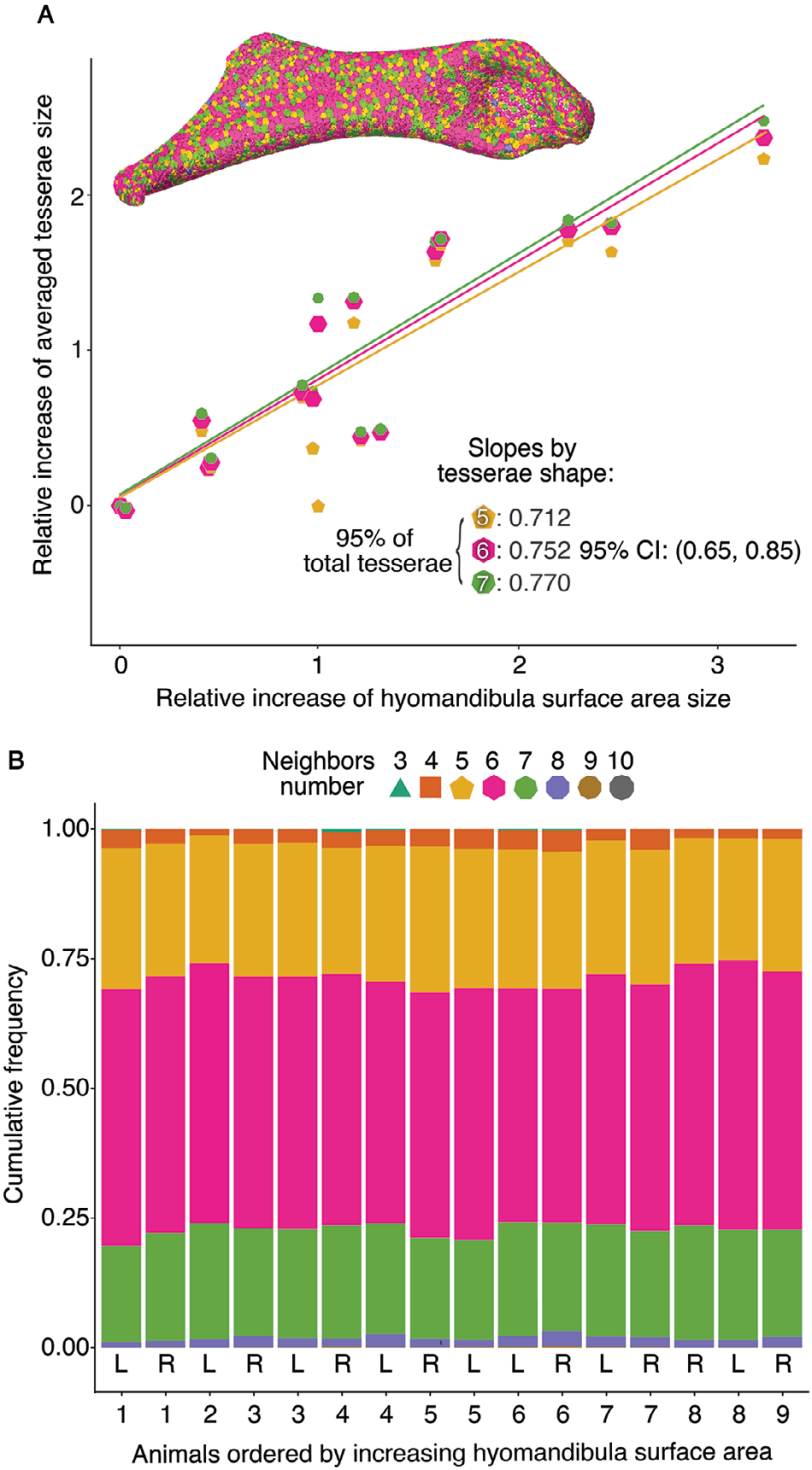
Tesserae shapes and their growth. A) The average relative increase in area size of the most common tesseral shapes (pentagons, hexagons, heptagons) as a function of hyomandibula surface area growth, both axes rescaled according to the value for the youngest ray investigated (1.3 years old). Regression lines plotted on these axes for the most common tesseral shapes have similar slopes, suggesting that the most common tesserae shapes experience similar increase in size across ontogeny, with different geometries equally contributing to the growth of the skeleton. B) Approximately 50% of the tesserae are hexagonal. This result is consistent with expectations of tiling geometries for predominantly flat surfaces (see text and Figure S3, Supporting Information). The increase in surface area from these three most common tesserae shapes is 25% slower than that of whole *hyomandibula* surface area. The occurrence frequencies of different tesserae geometries across all 16 specimens are strikingly consistent, with pentagons, hexagons and heptagons accounting for ≈95% of the total number.

The consistent number of tesserae in the *hyomandibula* tessellation speaks to a process whereby the growth of thousands of individual tesserae is coordinated to maintain a changing (but overall continuous and self‐similar) surface covering during skeletal growth. This calls for some type of regulation, perhaps through interaction with neighboring tesserae and the underlying cartilage. It has been proposed that the cells within and between tesserae could act as strain sensors, registering loading conditions in the skeleton and coordinating growth responses as a result (e.g., depositing more mineralized tissue in regions of higher load).^[^ ^3^, ^8^, ^14^ ^]^ We therefore explored the distribution of tesserae shape and size to determine if growth is progressing in predictable ways (e.g., according to aspects of tesserae or skeletal morphology), which could in turn point to regulating factors involved in skeletal growth and tessellation pattern generation. We evaluated each *hyomandibula* tessellation using 3D image processing, representing the tessellation as a two‐dimensional network of interconnected nodes (tessera centers) and edges (linear elements linking the nodes of neighboring tesserae) (Figure 6C). Based on the edges connected to each node, the network map yielded quantitative measures of tessera size and geometry, as well as of the patterns of tesserae connections (i.e., tessera shape distribution map) (**Figure** **3**; Figure S2, Supporting Information). With these data, the tessellations in our *µ*CT‐scanned datasets could then be characterized in bulk and rendered to illustrate patterns of variation of specific variables (e.g., color‐coding tesserae according to measurements of size or shape; Figure 3)

We found that, as with tesseral number, the distributions of tesserae in‐plane‐geometries were impressively consistent across age (Figure 3B), with six‐sided tesserae dominating, followed by five‐ and seven‐sided shapes (hexagons, pentagons and heptagons, respectively; Figure S1C,D, Supporting Information). Hexagons are an exceptionally common architectural motif in natural architectures,^[^ ^9^, ^15^ ^]^ permitting a complete uniform tiling of a surface by a regular shape that is both space‐efficient and structurally robust.^[^ ^16^ ^]^ Other geometries were occasionally present (e.g., from three‐ to ten‐sided tesserae), but were considerably less common. Regressions of tessera size (of the most common geometries) against *hyomandibula* surface area had consistent scaling coefficients, regardless of tesserae geometry (Figure 3A). This indicates that most tesserae are tracking the growth rate of the *hyomandibula*, although the growth rate of the dominant tesserae geometries (hexagons, pentagons, and heptagons) does lag somewhat behind that of *hyomandibula* surface growth (i.e., tesserae slopes are <1). This slight disconnect may be a function of the occurrence of tesserae with rarer shapes or incipient tesserae appearing within the tessellation, although our data argue that the growth of the *hyomandibula* surface is overwhelmingly due to the growth of tesserae that are already there.

At the same time, however, the local in‐plane tesseral topologies (i.e., the particular local constellation of tesserae geometries) differed from one *hyomandibula* to another (Figure S1C,D, Supporting Information). As a result, across the many *hyomandibulae* studied, specific anatomical features (e.g., the lateral fossa,^[^ ^17^ ^]^ where muscles attach, Figure 1; Figure S1D, Supporting Information) were crafted by entirely different tessellation patterns. This indicates that, whereas *hyomandibulae* from different animals and ages possess a generally identical diversity of tesserae (i.e., similar numbers of five‐, six‐ and seven‐sided tesserae), the exact spatial arrangements of those tiles varies. The statistically consistent, but topologically variable tessellations in stingrays contrast other examples in nature, such as the tessellated armor of boxfish, where patterns are not only largely comparable across individuals, but also left‐right symmetrical.^[^ ^9b^ ^]^ This indicates a stochastic rather than a predetermined generation of the pattern, similar to the emergent patterns seen in reaction‐diffusion systems,^[^ ^18^ ^]^ suggesting instead that the initial development of tesserae involves a random spatial distribution of tesserae growth centers over the *hyomandibula*’s surface.

Truly flat surfaces (i.e., that can never be “closed” around a volume) can be tiled completely by hexagons, as demonstrated by the familiar honeycomb of bees.^[^ ^19^ ^]^ However, for a closed surface (e.g., one without boundary) covered with tiles, heptagons, and pentagons are necessary to sculpt convex and concave surface contours, respectively. To describe the relationship of tile shapes on a closed surface, Euler famously derived the relationship between a tessellation's vertices (*V*), edges (*E*), and faces (*F*) as *V* – *E* + *F* = 2.^[^ ^20^ ^]^ For a surface fully covered with pentagons, hexagons, and heptagons only, this relation implies that the difference between the number of pentagons and hexagons should be exactly 12.^[^ ^20^ ^]^ In other words, in the special case where there are no heptagons (i.e., only hexagons and pentagons), a closed surface would require 12 pentagons and hexagons, as with fullerene structures.^[^ ^21^ ^]^ Given the large number of tesserae covering the *hyomandibula* (several thousand per skeletal element), if the stingray tessellation were to satisfy Euler's relationship, the number of heptagons and pentagons should be roughly similar (i.e., a difference of only 12 tesserae). We have shown, however, that there are appreciably more pentagonal than heptagonal tesserae (Figure S4, Supporting Information), which should not be possible with a closed Eulerian tessellation. One possible explanation for the departure from Euler's equation is that the *hyomandibula* is almost, but not fully covered by tesserae (Figure S3 middle, Supporting Information). Additionally, our comparison to Euler's theorem assumes idealized geometric shapes—perfect pentagons, hexagons, and heptagons—which do not account for the irregularities or imperfections in tile geometry that occur in the unmineralized intertesseral regions. We also note that along anatomical ridges, tesserae are often not flat polygons but rather somewhat curved, which is in breach of Euler's assumptions (see Figure S5, Supporting Information) and may explain the comparative lack of heptagons (which introduce negative curvatures when added to hexagonal tessellations). While Euler's formula provides a useful mathematical framework, its application here must be understood with the caveat that biological systems often exhibit variability and imperfections not fully captured by pure geometric approximations.

Interestingly, exceptions to the typical tessellated cartilage strategy of building with numerous small and flat tiles (Figure S1B, Supporting Information) were consistently observed (in all specimens) in association with specific anatomical features. In these areas, the local curvature and irregularity of the tesserae were more pronounced, particularly in regions with a high degree of curvature.^[^ ^22^ ^]^ The local curvature of the *hyomandibula* was instead a function of the surface geometries of fewer, comparatively large tesserae (Figure S1C,D, Supporting Information). For example, massive bulbous tesserae dictated the *hyomandibula*’s small radius of curvature along the crests of the anatomical ridgelines (Figure S1C,D, Supporting Information), similar to large keystone bricks in an archway. Additionally, at the margins of the medial fossa (Figure 1), odd hourglass‐shaped tesserae were occasionally seen bridging the juxtaposed medial and lateral tesseral layers (Figure S6, Supporting Information). These regions of large curvature and local irregularity, often associated with ridges, are critical for understanding the biomechanical role of tesserae in maintaining structural integrity under varying stress.^[^ ^22^ ^]^ Moreover, this is consistent with observations of microscale “tesselle” packing in bone, another biological tessellation,^[^ ^23^ ^]^ where similar 3D arrangements of irregular boundaries, organic gaps, and connecting collagen fibrils were observed. This further highlights the limitations of purely geometric models in describing biological structures.

## 4 Maintaining a Tessellation

**Figure 4:**
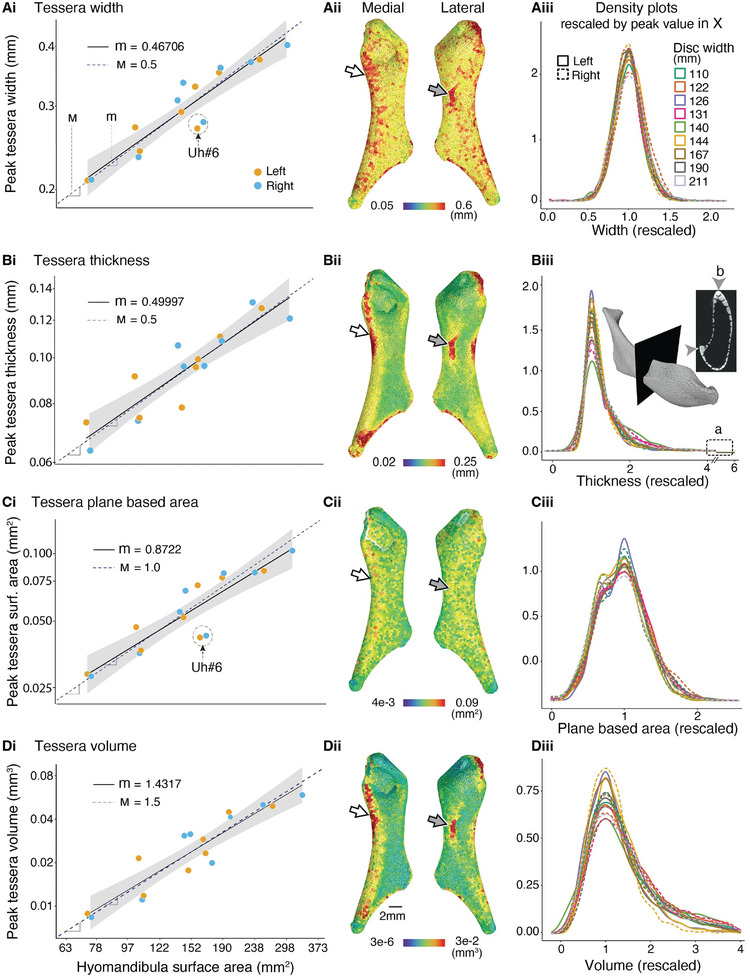
Tesserae dimensions and self‐similar growth over ontogeny. Measured tesserae morphometric variables are shown in figure rows A) thickness, B) width, C) plane‐based area, and D) volume. Morphometric changes over ontogeny are shown as peak‐value regressions relative to *hyomandibula* surface area (i, left graphs) and whole‐*hyomandibula* distributions (including all tesserae from each *hyomandibula*), rescaled so that all distributions have their peak value at 1 (iii, right graphs). The highly overlapping graphs (iii) of rescaled tessera width and plane‐based area, together with isometric growth of their peak values (i), indicates that tesserae have self‐similar growth patterns and are growing isometrically with the skeletal surface. Surface rendering of an exemplar *hyomandibula* with tesserae color‐coded (ii; green to red, increasing values for each variable) are shown in the middle column. Note the anatomical locations of reddish regions in the renderings, often associated with ridges on the *hyomandibula* (white arrow = ventral ridge, gray arrow = crest bordering lateral fossa; see Figure 1). Note also the overlap of distributions is perhaps less precise for the thickness distributions on the right, suggesting other growth laws may be at play there. In the left graphs (i), both horizontal and vertical axes are logarithmically scaled, and the horizontal axes from Ai to Ci are shared with Di. The symbol “m” denotes the isometric growth slope (dashed lines), while “m” represents data regression slopes (solid lines).

To understand how tessellation rules vary with age, we examined several tesserae morphometric variables, including width, thickness, surface area, and volume (**Figure** **4**), rescaling the dataset distributions by their peak values to overlap at their maxima. Peak values for all four tesserae morphometric variables were highly linearly related with animal size (Figure 4A‐i–D‐i), illustrating that as animals age, tesserae do become thicker and wider (and, as a result, show higher values for area and volume). These observations on whole skeletal elements support similar trends seen in previous works on 2D cross‐sections or partial skeletal elements.^[^ ^12^, ^24^ ^]^ On average, the tesserae we observed approached the resolving limit of the human eye, with those of the largest animal (≈400 µ m wide, 120 µ m thick) about two times the width and thickness of those of the smallest (Figure 4A,B‐i,ii). In color‐coded spatial maps of the four morphometric variables (Figure 4‐ii), there are no large‐scale patterns to the arrangements of tesserae, although wider and thicker tesserae with larger volumes (Figure 4A,B‐ii, arrows) tended to be associated with the anatomical ridgelines (Figure 1), as mentioned previously. Although ridgeline tesserae tended to be thicker (Figure 4B‐ii), the fact that they did not also have particularly large surface areas (Figure 4C‐ii) suggests that they may simply represent asymmetric in‐plane geometries (i.e., wider in one dimension). Moreover, when comparing *hyomandibula* tesserae to uniform polygons, it can be seen that tesserae are not perfectly regular in shape (Table S1 and Figure S1A, Supporting Information).

In contrast to tesseral width and area, the distribution of tesserae thicknesses is far broader, with the thickest tesserae being ≈10x that of the thinnest (Figure 4B‐iii x‐axis is truncated for visual clarity, values at region **a** in the graph possibly indicating the thickest tesserae positions at **b** in the inset *µ*CT scan). Furthermore, the particularly high correspondence between the spatial maps of tesserae thickness and volume (Figure 4B‐ii,D‐ii) compared with the poor correspondence with surface area (Figure 4Cii) identifies the out‐of‐plane dimension (thickness) as the main component driving the variation in tessera volume, indicative of relatively constant in‐plane dimensions (surface area, width). Although the main peak is mostly due to hexagons, an extra maximum is visible in the rescaled tesserae area distribution (Figure 4C‐iii). This is due to the presence of pentagons that are generally smaller than hexagons with the same edge length. Since the number of heptagons is much smaller, only a weak shoulder is generated on the right side of the main peak (because heptagons with the same edge length are larger). No such extra maxima are seen in the tesserae width distribution Figure 4A‐iii, likely because the difference in width between hexagons and pentagons is not large enough, compared to the full width at half maximum of the size distribution, for such a peak to become visible. This was verified by checking that the separate distributions of hexagons and pentagons have only a single maximum in all cases (data not shown).

Different factors may therefore regulate or constrain in‐plane and out‐of‐plane growth (e.g., thickness should be less constrained by surface topography). The particularly massive tesserae found on the anatomical ridgelines and in regions of muscle attachment (Figures 1, 2, 4 reddish areas, 4Biii inset) further support our hypothesis that tesserae with in‐built surface curvature (see above) tend to form in areas subject to particularly high mechanical stress, since the thickness of the tessellated shell should be a deciding factor in the bending stiffness of the *hyomandibula*. The disproportionately thick (reddish color in Figure 4B‐ii) tesserae on the ridgelines likely also indicate the high mechanical stress resulting from muscle attachment. In contrast, we argue below that the in‐plane growth of the stingray tessellation is coordinated by geometric factors that are consequences that cascade downstream from the random initial patterning of tesserae growth centers.

## 5 Growth Rules and Geometric Considerations

The size‐rescaled datasets of tesserae morphometric variables are strikingly similar across the numerous *hyomandibulae* sampled, with tesserae width and area distributions overlapping especially well (Figure 4A,C‐iii). This indicates that tesserae in‐plane dimensions are relatively invariant once mean size is removed as a factor, suggesting a surprisingly self‐similar growth process in tessellated cartilage. Curiously, this echoes well‐known self‐similar growth processes for distributions of grain size in heat‐treated metals^[^ ^25^ ^]^ and precipitate size in tempered alloys.^[^ ^26^ ^]^ Self‐similar size distribution of droplets or precipitates also occurs during Ostwald ripening, where large particles grow at the expense of smaller ones, thereby keeping the overall particle volume constant.^[^ ^27^ ^]^ In contrast to this, all tesserae—large or small—keep growing, since there is apparently no resorption of mineralized tissue in sharks and rays, which obviously implies that the overall tesserae volume does not stay constant.

**Figure 5:**
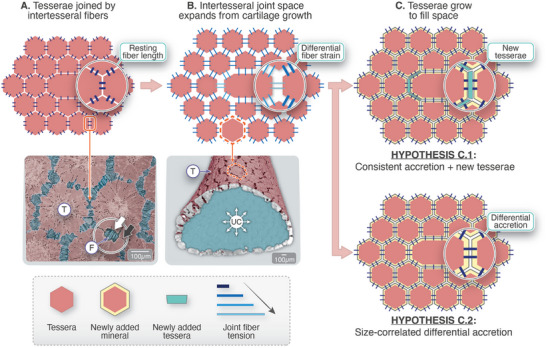
Two possible growth scenarios for a tessellation with non‐identical tiles. A) Schematic 2D tessellation in original static situation, with the original tiles in coral red, linked by thick blue fibers with resting tension. Below, an in‐plane section through a SR‐*µ*CT of a stingray *hyomandibula* shows a similar arrangement (T: tessera, F: fibers). Note the areas of contact (black arrow) and fibrous connection (white arrow) in the intertesseral joints. B) When the unmineralized cartilage core (UC) of the skeleton expands during growth (illustrated in the *µ*CT volume rendering below), homogeneous expansion of the plane would result in tesserae pulling apart from each other, variably loading the fibers between them according to the size of neighboring tiles (as indicated by different fiber colors). C) Two hypotheses of mineral growth are presented to recover the space which growth introduces into the tessellation: (C.1) if new mineral (yellow tissue) is deposited on tesseral edges at a constant rate, this will not be adequate to fill all introduced gaps (e.g., next to larger tesserae) and new tesserae (in venice green) will have to be added. (C.2) However, if local apposition rate is proportional to tesserae size (e.g., as a function of local fiber strain), the tessellation can continue to grow isometrically with the same number and pattern of tiles, as a continuous surface. For information about the SR‐*µ*CT data, see Seidel et al. 2016.^[^ ^8^ ^]^

Whereas growth laws in metal grains and precipitates are governed by thermodynamics,^[^ ^28^ ^]^ biological and/or physicochemical processes are likely at play in tessellated cartilage. Tesserae are believed to grow along their margins, where they exhibit complex interactions with neighboring tesserae^[^ ^12^, ^29^ ^]^ (**Figure** **5**A). In these narrow intertesseral joints —which function like ultrastructural skeletal sutures— tesserae show local zones of close contact (Figure 5A *µ*CT, black arrow) and gapped fibrous zones (white arrow), where organic fibers tether tesserae to each other.^[^ ^12^, ^14^, ^30^ ^]^ Cells are densely packed between the linearly arrayed joint fibers^[^ ^2^, ^8^, ^31^ ^]^ and these become incorporated into the tesserae (see black lacunae spaces; Figure 5A *µ*CT), as tesserae accrete mineral at their edges,^[^ ^12^, ^14^, ^30^ ^]^ The factors regulating this appositional growth are unknown, but the localization of mineralization factors along the margins of tesserae indicate they are indeed active growth zones.^[^ ^32^ ^]^

The most natural assumption is that, as a stingray skeletal element grows (by expansion of its unmineralized cartilage core; Figure 5B), tesserae accommodate this growth by apposition of new material at a constant rate along their surfaces. In a tessellation consisting of identical tiles (e.g., regular hexagons), this would indeed lead to an isometric expansion of the tiled surface, maintaining a continuous skeletal covering. However, when there is a size distribution of tiles —as we have demonstrated in the stingray *hyomandibula*— a geometric incompatibility would occur with this growth process. This is exemplified in Figure 5B where a large tessera is embedded into a hexagonal tiling, as an example. When a new layer of constant thickness is added to each tessera (Figure 5, yellow layer), a gap would still gradually appear in the tiling adjacent to the larger tile (i.e., the space introduced next to larger tiles is larger). We propose two possible scenarios to mitigate this effect: in the hypothesis in Figure 5C.1, small new tesserae (shown in green) would be introduced to fill the gaps. Such a process would obviously increase the number of tesserae during growth of the *hyomandibula*, which is in contradiction to our observations in the stingray system here (Figure 2D).

In the alternative hypothesis depicted in Figure 5C.2, the apposition rate is larger on the surface of the larger tessera (note the thicker yellow swath in the insets in Figure 5C.2 vs 5C.1). The consequence of such a model is that tesserae would accrete new tissue in proportion to their size. This hypothesis —where larger tiles grow faster— corresponds to a model of self‐similar growth that is in perfect correspondence with the scaling of the size distributions shown in the right panels of Figure 4. The scenario depicted in Figure 5C.2 is, therefore, in full agreement with the observations of tesserae growth in the stingray *hyomandibula*. Moreover, the link between tessera size and growth rate indicates that the original size of a tessera will determine its speed of growth for the animal's entire lifetime, underlining that the particular locations of initial growth centers are vital determinants of later skeletal form and tessellation pattern.

The growth scenario in Figure 5C.2 being the most likely process occurring in tesserae growth raises the question of how such a non‐uniform apposition rate on tesserae might be biologically controlled. Based on the anatomy of intertesseral joints and our growth hypothesis, we speculate that a mechanobiological regulatory mechanism is likely driving appositional growth of tesserae, reminiscent of how teeth can be moved within the jaw bone of mammals.^[^ ^33^ ^]^ As with intertesseral fibers linking tesserae,^[^ ^2b^ ^]^ the roots of teeth are anchored via a collagenous periodontal ligament into the jaw.^[^ ^33^, ^34^ ^]^ When teeth are loaded, the stress on these fibers activates cells residing in the periodontal ligament to add new bone to the tooth socket in those regions where fibers are tensed more, while bone is simultaneously removed from the contralateral side of the socket.^[^ ^33^, ^35^ ^]^ Collagen turnover is vital to this process and varies locally within the periodontal ligament, being higher closer to the bony surface.^[^ ^36^ ^]^ While in orthodontics there is a removal and addition of bone,^[^ ^37^ ^]^ no resorption is needed for the growth of tesserae, where only a mechanically controlled addition of mineralized tissue is required. Determining the nature of cellular involvement^[^ ^38^ ^]^ and rates of collagen turnover^[^ ^39^ ^]^ in the intertesseral joint space would pinpoint key functional drivers of this responsive growth mechanism.

Assuming that cells in the fibrous tissue connecting tesserae are mechanosensitive and stimulated to deposit mineralized tissue when the fibers are challenged in tension, we hypothesize that Figure 5C.2 would develop in a self‐organized way during skeletal ontogeny. As the skeleton develops, the cartilage expanding beneath the tessellation would increase its surface area (Figure 5B), creating tensile stresses in the interstices of the surface tessellation, straining the intertesseral fibers. The purely geometric aspects of growing a tiled surface with variable tile size would inherently create a mosaic of fiber stresses in the tessellation: the larger intertesseral gaps introduced adjacent to larger tiles as the cartilage substrate grows would result in comparatively greater stresses on those fibers and, therefore, stimulate increased formation of mineralized tissue on the surfaces of the larger tesserae.

Since forces would be equal on both ends of a fiber stretched between two tesserae, growth rates will be the same on opposing faces of neighboring tesserae, while they might be different on different faces of one tessera, depending on the size of the gap that develop with respect to each neighbor. The equipartitioning of the space between neighboring centers is known as Voronoi tessellation.^[^ ^40^ ^]^ Therefore, after sufficient growth, any starting configuration of initial growth centers would approach a Voronoi tessellation. If the skeleton's earliest tessellation is the blueprint followed for the animal's lifetime, the factors driving the spatial partitioning of different tesserae types —flat versus highly‐curved morphologies— could be a function of a minimum size constraint acting on nascent tesserae early in development. Tesserae apparently first form in association with small cell clusters^[^ ^8^, ^41^ ^]^ and although *hyomandibulae* grow isometrically (Figure 2), the cells in the cartilage do not change drastically in size.^[^ ^12^ ^]^ This could indicate that early cell clusters (i.e., incipient tesserae growth centers) have a threshold minimum size. Given this and our evidence from the current work that growth centers are distributed randomly, cell clusters that happen to arise further from others would have more room available to grow and to connect to a larger number of neighboring growth centers. We would expect then that tesserae with more sides/neighbors would tend to be inherently larger than those with fewer neighbors. This is indeed supported by our data (Figure S7, Supporting Information). Additionally, a minimum threshold size for early cell clusters would mean that the regions of highest curvature (e.g., anatomical ridges), might only have enough surface area to support a single growth center, resulting in single tesserae which are responsible for crafting ridgelines throughout life. This constraint would have profound effects on how strongly curved regions are built in tessellated cartilage, but may also be relevant for tissue biomechanics, as the ridges likely provide important buttressing against bending loads during jaw movements (Figure 1B).

Intertesseral cell mechanosensitivity has yet to be demonstrated, but the close association of cells with tethering fibers in the joints, and the persistence of communicating vital cells in tesserae, argue the cells may orchestrate growth in response to mechanosensory input.^[^ ^8^, ^14^ ^]^ Intertesseral cells are at least morphologically similar to chondrocytes, which are known in mammals to synthesize both collagens and collagen‐cleaving enzymes^[^ ^42^ ^]^ (e.g., matrix metalloproteinases, MMPs). Since intertesseral joint fibers are at least partly collagenous,^[^ ^2^, ^43^ ^]^ cells in the joint region could therefore be responsible for coordinating both inorganic and organic architectural components during skeletal growth. As the metabolism of cartilage is comparatively low,^[^ ^44^ ^]^ only a few cells per joint would be adequate to effect this elegant growth system; this is supported by histological observations of cell density in the joint space,^[^ ^2^, ^8^, ^14^, ^31^ ^]^ although work is needed to characterize the activity and synthesis of joint cells and the loads they experience in vivo.

## 6 Conclusion

We have shown that stingray cartilage solves the problem of growth of a mineralized surface inherently incapable of remodeling by breaking the crust into tiles connected by collagenous fibers. Self‐similar growth, as observed experimentally, may occur based on a simple growth rule where the straining of these fibers controls the apposition rate to the polygonal tesserae by means of mechanosensitive cells sitting between these fibers. In this way, the integrity of the tiling can be preserved at all times during growth without the need for an overall control of the mineral apposition rates, provided cells in the interstices are mechanosensitive and deposit mineral in response to strain. The overall tiling will then be close to a Voronoi tessellation that has self‐similar properties across ontogeny. Although details of the biological control mechanisms still need to be elucidated, the ultrastructural morphometric analysis performed here clarifies critical geometric conditions and growth laws for the seamless growth of a tessellated body, lessons that reach far beyond the biological system studied.

## 7 Experimental Section

### Specimens and Sample Processing

A total of 16 *hyomandibulae* (Figure 2; and Table S2, Supporting Information) were collected from nine individuals of *Urobatis halleri* (Haller's round ray; Urotrygonidae, Batoidea), caught for another study^[^ ^48^ ^]^ using beach seine nets in San Diego and Seal Beach (California, USA). *Urobatis halleri* was chosen as a study animal due to its comparatively small body size, making the *hyomandibula* and its tesserae apt for high‐resolution scans in both laboratory settings and SR‐*µ*CT scanners. There is also a relatively large amount of information available on this species’ skeletal biology, it having been the subject of considerable developmental and structural research (e.g.,^[^ ^8^, ^16^, ^17^, ^20^, ^25^, ^46^, ^49^ ^]^). Sampled individuals were balanced regarding males (*n* = 4) and females (*n* = 5), and covered various developmental stages, including yolk sac embryos (1.3–11 cm DW), juveniles (11–15.5 cm DW), and adults (>15.5 cm DW; Figure S1, Supporting Information).^[^ ^10^ ^]^ Specimens were shipped on dry ice and stored at −20 °C.

The *hyomandibula* was chosen as a study element for several reasons. As mentioned in the Introduction, the *hyomandibula* is a manageable size for dissection and scanning, while still having thousands of tesserae for quantification, bearing also several distinct local concave and convex anatomical features for investigating how tesserae manage curvatures. Additionally, the *hyomandibula* is also an integral component of the stingray's cartilaginous skeleton, playing an indispensable role in jaw mobility by connecting robustly to the jaws and forming a hinge‐like joint with the cranium. To extract the *hyomandibulae*, animal specimens were thawed in water, *hyomandibulae* dissected out, and immediately preserved in 70% ethanol. Both right and left *hyomandibulae* were dissected from each animal, with the exception of two individuals where only one intact *hyomandibula* could be obtained (red *hyomandibulae* in Figure 2).

### Micro‐Computed Tomography

**Figure 6:**
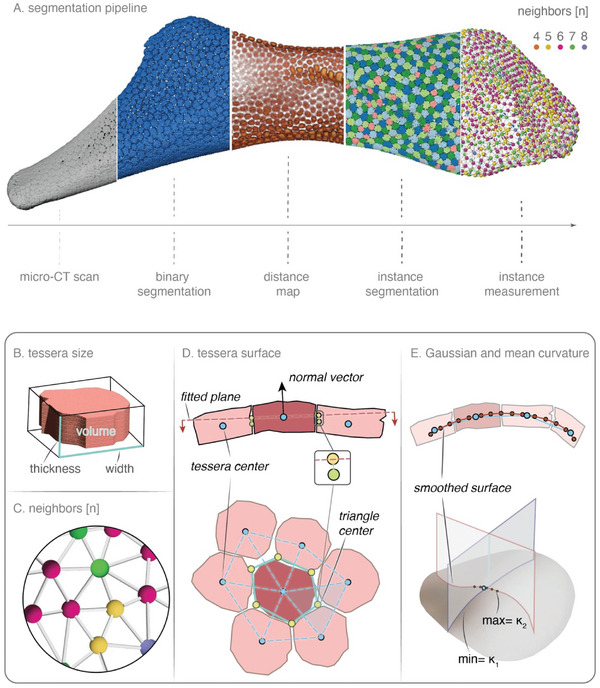
Segmentation pipeline and variables measured. A) Amira pipeline for quantifying stingray tessellations. Morphometric measurements of individual tesserae acquired through the Amira quantification pipeline included B) tesserae size, volume, thickness and width. C) To quantify each tessera's relationship with its neighbors, the tesseral network was rendered in Amira, with nodes representing tessera centroids and the color of the node representing the number of neighboring tesserae (indicated by connecting lines). D) Tesserae surface area was quantified by defining tesserae centers (blue dots), then drawing triangles to connect those (blue dashed lines) and determining those triangles’ centroids (green dots), which were then projected (yellow dots) onto a fitted plane (red dashed lines) perpendicular to the central tessera's normal vector (black arrow). The projected points (yellow dots) were then connected by polylines to define the vertices approximating tesserae surface geometry (venice green), allowing calculation of tesserae surface area. E) Gaussian and mean curvatures were quantified by fitting a finer‐meshed surface (red dots) to the coarser surface defined by the tesseral centers (blue dots). A tessera's local Gaussian/mean curvatures were defined based on that smoothed surface, at the location of the smoothed surface node (red) nearest to that tessera's center (blue).

*Hyomandibulae* were imaged using high‐resolution micro‐computed tomography (*µ*CT) (**Figure** **6**A), with a final voxel size of 4.9 µm: 12 samples were imaged using a Bruker *µ*CT (Skyscan 1172) at 60 kV source voltage, 167*µ* A source current (for additional details^[^ ^2^, ^8^ ^]^), while four samples were scanned using an RX Solutions EASYTOM *µ*CT at 100 kV source voltage, 100*µ* A source current. Reconstruction was performed using associated software from each scanner. *Hyomandibulae* were scanned mounted upright in inverted centrifuge tubes, with ethanol‐soaked Kimwipes in the caps to preserve hydration during scanning and avoid collapse of the tessellated layer (occurring as unmineralized cartilage dries).

### 3D Imaging Reconstruction, Segmentation, and Analysis

Image stacks reconstructed from scanner software were rendered, segmented, and analyzed using the extended version of the AmiraZIBEdition software (Thermo Fisher Scientific, Bordeaux, France, and Zuse Institute Berlin, Germany, package version 2020, 2022.06, and 2022.07), leveraging established semi‐automatic segmentation pipelines and algorithms^[^ ^9a,c^ ^]^ quantification of *hyomandibula* tessellations in their entirety (Figure 6A). The development of high‐resolution visualization techniques, especially tomography (Figure 6A Panel 1), had enabled a more in‐depth exploration of intricate structural components. However, the quantification of specific geometric characteristics remains a complex, time‐intensive endeavor for systems with large numbers of independent elements in close contact (e.g., tesserae). The workflows employed facilitate high‐throughput instance segmentation,^[^ ^45^ ^]^ assigning instance‐level labels to different objects of the same class, enabling the quantification of diverse elements^[^ ^9^, ^46^ ^]^ (e.g., tesserae or voids within them) within 3D grayscale images.

To briefly summarize the segmentation process, in each *hyomandibula* dataset, the tesseral layer was first segmented relative to background using grayscale thresholding. The segmentation was then cleaned of extra‐ and intra‐tesseral noise (e.g., lacunar gaps inside tesserae), resulting in a binary dataset, retaining only mineralized tissue (tesserae) and background. A 2D Euclidean^[^ ^47^ ^]^ or random‐walk distance transform^[^ ^9^, ^48^ ^]^ was applied to the tesseral layer (segmented foreground) and the resulting distance map used as the input for a hierarchical watershed segmentation,^[^ ^9^, ^49^ ^]^ to isolate individual tesserae from one another. This approach provided a high‐throughput segmentation of the thousands of tesserae covering each *hyomandibula* and significantly reduced manual adjustments. Typically, a small number of tesserae (<10% of 3000) were not accurately identified (e.g., two tesserae labeled as one or vice versa, Figure S1E, Supporting Information) or not clearly captured (e.g., the very thin, partially‐formed tesserae fringing the medial fossa, Figure S1F, Supporting Information). The former errors were corrected through semi‐manual proof‐reading tools in Amira specifically developed for this purpose, whereas the latter (partial tesserae) were removed from the analysis, considered to result from compounding effects of low mineralization and scan resolution.

The segmentation of nearly all tesserae covering the *hyomandibula* surface enabled bulk measurement of various morphometric variables (Figure 6B–E) for all individual tesserae, using the following methods. The centers of tesserae were localized by averaging the coordinates of all voxels within each tessera. From these centers, a region adjacency graph (RAG) (see Figure 6A Panel 5 and Figure 6C) was constructed using the “Create Region Adjacency Graph” module. The RAG represents each tessera by a node at its center, connected to neighboring centers by linking edges (linear segments) (see Figure 6C). In this way, the RAG acted as a “neighbor graph” to quickly visualize associations among tesserae, allowing also easy proofreading, including the removing of erroneous edges or the adding of extra edges (e.g., if two tesserae were clearly anatomical neighbors from the raw data, but did not appear to touch in the segmentation, due to lower mineral density at their margins). The RAG was used to represent tessera geometry as a function of **tessera neighbor number** (see Figure 6A Panel 5 and Figure 6C), determined by the number of edges connected to a tessera's central node (e.g., hexagonal tesserae in the RAG are connected by six edges to six neighbors) (Figure 6C). **Tessera thickness (**Figure 6B) was quantified by enclosing each segmented tessera in a minimal cuboid (i.e., bounding box) and measuring the side length in line with the thickness axis (i.e., the outward‐facing normal vectors established from tessera centers). To accurately determine the thickness axis and hence the orientation of each tessera (i.e., its inward and outward faces), a signed distance map^[^ ^9c^ ^]^ was utilized, measuring the distance from all voxels to the nearest tessera‐background interface voxel, resulting in negative versus positive values inside versus outside the skeleton, respectively. Starting from each tessera center point and summing the signed distances along the six directions defined by the normals of the cuboid faces, the direction that resulted in the maximum summed distance value represented the outward facing direction. This method proved robust, even for tesserae of odd shapes or those containing background voxels^[^ ^14^ ^]^ (i.e., cellular lacunae). **Tesseral width (**Figure 6B) was quantified using the longest bounding box dimension that was not the thickness dimension. This helped to avoid conflating tesseral thickness and width in the case of the columnar tesserae found in curved regions,^[^ ^2^, ^24^ ^]^ which were taller than wide. **Tessera volume (**Figure 6B) was calculated by multiplying the number of voxels within each tessera by the voxel size. **Tessera surface area** (Figure 6C) was calculated using a plane‐based approach. This method involves the constructed region adjacency graph that connects a given tessera center with the centers of adjacent tesserae. Each geometric area of tessera was then determined by forming closed polylines. These polygon vertices are the projected centroid of adjacent triangles/quads, which extend from the tessera's center to the centers of adjacent tesserae, onto the pre‐determined fitted surface. Lastly, to understand the relationship of tesserae to particular hyomandibula surface topologies, for each tessera center, the nearest point was identified on the iteratively smoothed hyomandibula surface (derived from the original RAG, Figure 6E upper), and used the principal curvatures of at that position to calculate **Gaussian curvature** and **mean curvature** of the *hyomandibula* surface in association with each tessera (Figure 6E lower). All data were analyzed and visualized using the R studio ggplot package.

## Conflict of Interest

The authors declare no conflict of interest.

## Author Contributions

Binru Yang helped with segmentation, analyzed, interpreted and visualized data, made figures, wrote the manuscript. Paul Zaslansky (P.Z.) performed *µ*CT scans. Daniel Baum (D.B.) and David Knötel (D.K.) designed the Amira segmentation pipeline and analyzed data and D.K. processed most segmentations. Jana Ciecierska‐Holmes, Jan Wölfer, and Mason N.Dean (M.N.D.) helped with data mining and data analysis. Júlia Chaumel helped with segmentation, figures, and Supporting Information. M.N.D., D.B., and P.Z. conceived the study, and M.N.D. and Peter Fratzl directly supervised the research, analyzed data, interpreted and visualized results, helped with figures, and wrote the manuscript. All co‐authors read and approved the manuscript.

## Supporting information



Supporting Information

## Data Availability

The data that support the findings of this study are openly available in Edmond at https://doi.org/10.17617/3.IAMK5U, reference number 1.
