# Supplementary material for: Growth of a Tessellation: Geometric rules for the Development of Stingray Skeletal Patterns
Source: Adv Sci (Weinh). 2024 Nov 7;11(48):2407641. doi: 10.1002/advs.202407641 (PMC11672314; doi:10.1002/advs.202407641)
Supplement: Supplementary file 1 — Supporting Information [file ADVS-11-2407641-s001.pdf]

ADVANCED SCIENCE
Open Access

Supporting Information

for *Adv. Sci.*, DOI 10.1002/advs.202407641

Growth of a Tessellation: Geometric rules for the Development of Stingray Skeletal Patterns

Binru Yang, David Knötel, Jana Ciecierska-Holmes, Jan Wölfer, Júlia Chaumel, Paul Zaslansky, Daniel Baum, Peter Fratzl and Mason N. Dean**

ADVANCED SCIENCE
Open Access

Supporting Information

for *Adv. Sci.*, DOI 10.1002/advs.202409326

Growth of a tessellation: Geometric rules for the development of stingray skeletal patterns

Binru Yang, David Knötel, Jana Ciecierska-Holmes, Jan Wölfer, Júlia Chaumel, Paul Zaslansky, Daniel Baum, Peter Fratzl, Mason N. Dean**

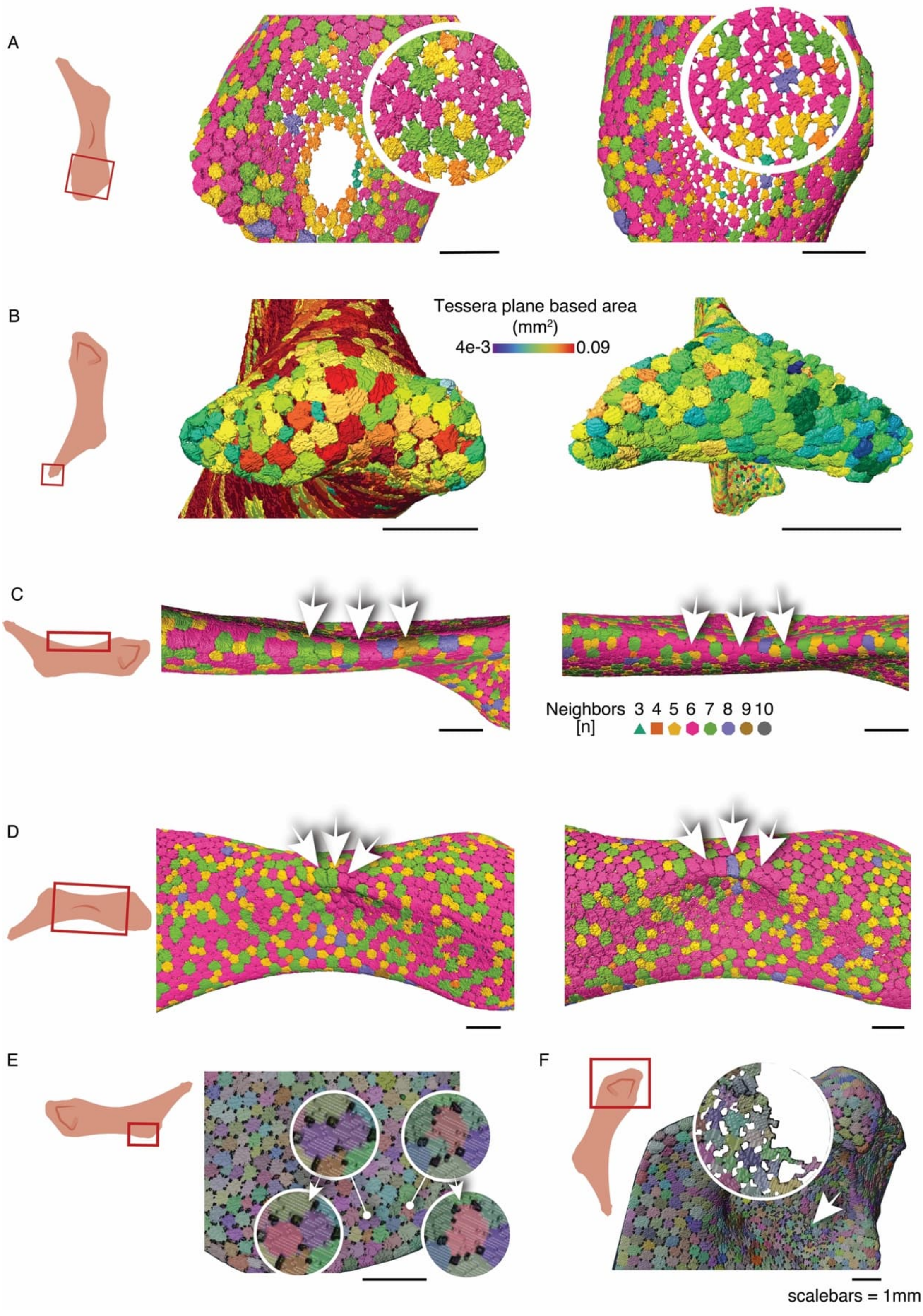

A
B
Tessera plane based area
(mm²)
4e-3
0.09
C
Neighbors 3 4 5 6 7 8 9 10
[n]
D
E
F
scalebars = 1mm

Supplementary Figure S1. Irregular tesserae and tesseral patterns are depicted as follows: (A) In the medial fossa, star-like tesserae are highlighted and color-coded according to the number of neighboring tesserae. (B) Tesserae at the distal end are color-coded by size, with dark blue representing smaller tesserae and red indicating larger ones. Note that, for the same anatomical region, the right image (from Uh#6) features an abundance of smaller tesserae; compare with data in Figures 2D and 4, showing this individual tended to have comparatively numerous and small tesserae. (C) Variations in local tesseral topologies observed among individual specimens; note the white arrows indicate comparable regions with very different neighbor numbers. (D) Tesserae along large lateral ridges are relatively large and exhibit greater length in one dimension compared to the tesserae in other regions. This particular ridge borders the lateral fossa (Figure 1C), an area where muscles attach to the hyomandibula (E) Segmentation errors (top circles) and their correction (bottom circles): the left circles illustrate under-segmentation of tesserae, the right show over-segmentation. (F) Thin and partially-formed tesserae fringing the medial fossa.

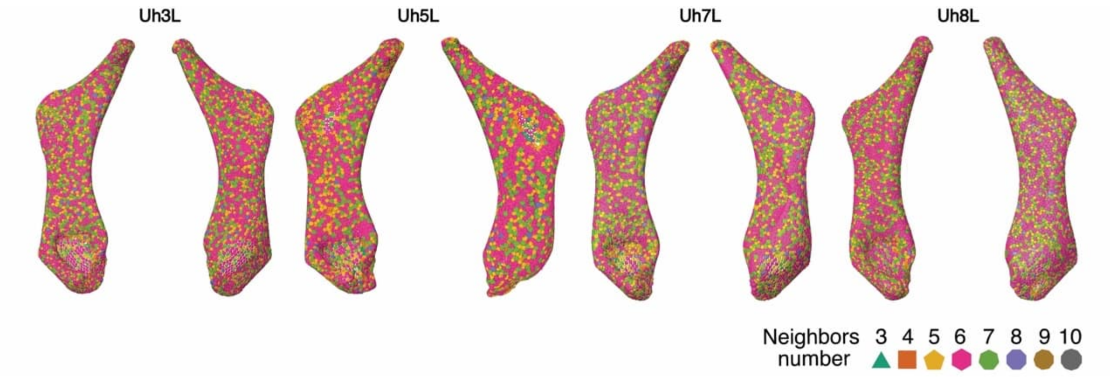

Supplementary Figure S2. *Hyomandibulae* Uh#3L, 5L, 7L, and 8L are presented in both medial and lateral views (left and right images, respectively, for each individual), with color-coding based on the number of neighboring tesserae. Note the dominance of hexagonal, pentagonal and heptagonal tesserae, but the lack of specific tesseral pattern in comparable anatomical locations across datasets.

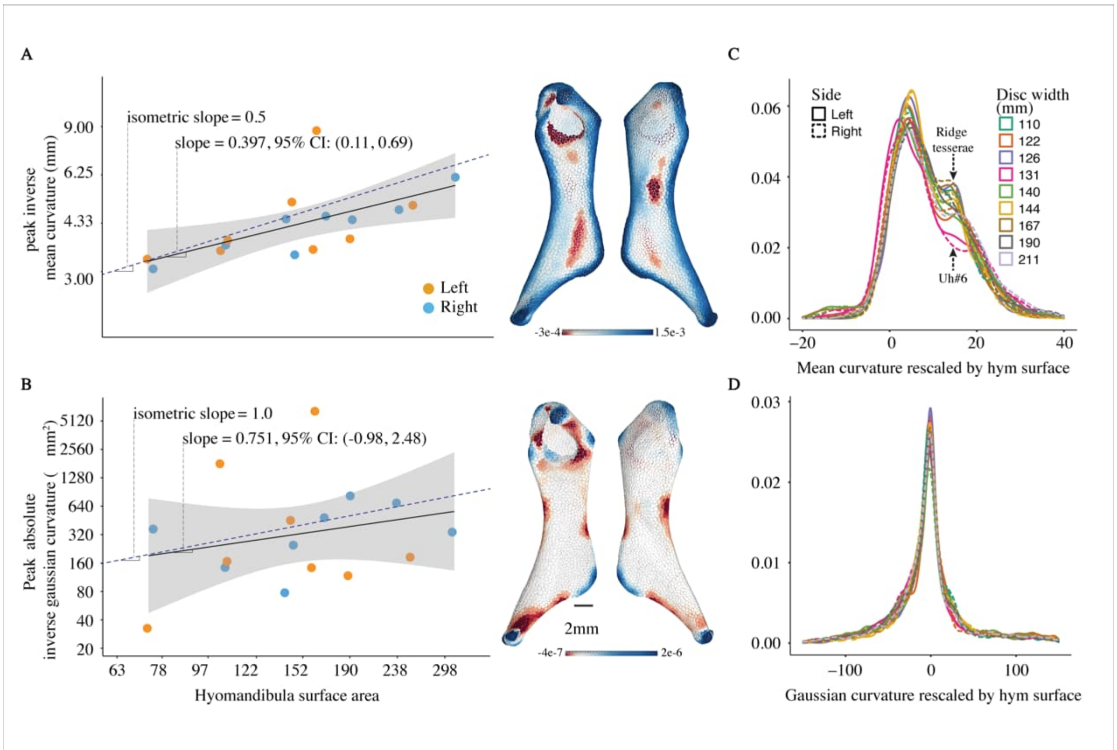

Supplementary Figure S3. *Hyomandibulae* local curvatures over ontogeny. We used the local minimum and maximum curvatures determined for each tessera (see Figure 6E) to calculate mean and Gaussian curvatures. To investigate how curvatures change with age, we plotted the peak x-values for the unscaled mean and Gaussian curvature distributions (not shown), against hyomandibula surface area (S3A and S4B, respectively), using inverse y units for a more intuitive isometric growth slopes (i.e. +0.5 for mean curvature and +1.0 for Gaussian curvature). We then scaled the curvature distributions according to hyomandibula surface area (see Figure S3C and D). The strong overlapping of all scaled mean and Gaussian curvature distributions, respectively, provides evidence that the *hyomandibula* grows isometrically and maintains its shape throughout ontogeny. Surface renderings of exemplar *hyomandibulae* with tesserae color-coded for curvature are shown in the middle of the figure, with red and blue representing negative and positive curvatures, respectively, and white indicating zero curvature surfaces. Note in these renderings and in S3C and D that most curvature values are concentrated near 0 for both mean and Gaussian curvatures, indicating that the *hyomandibula* consists of predominantly flat areas that enclose the cartilage volume. In keeping with Eulerian relationships, this result is also consistent with the finding that most tesserae are hexagonal (see Figure 3B).

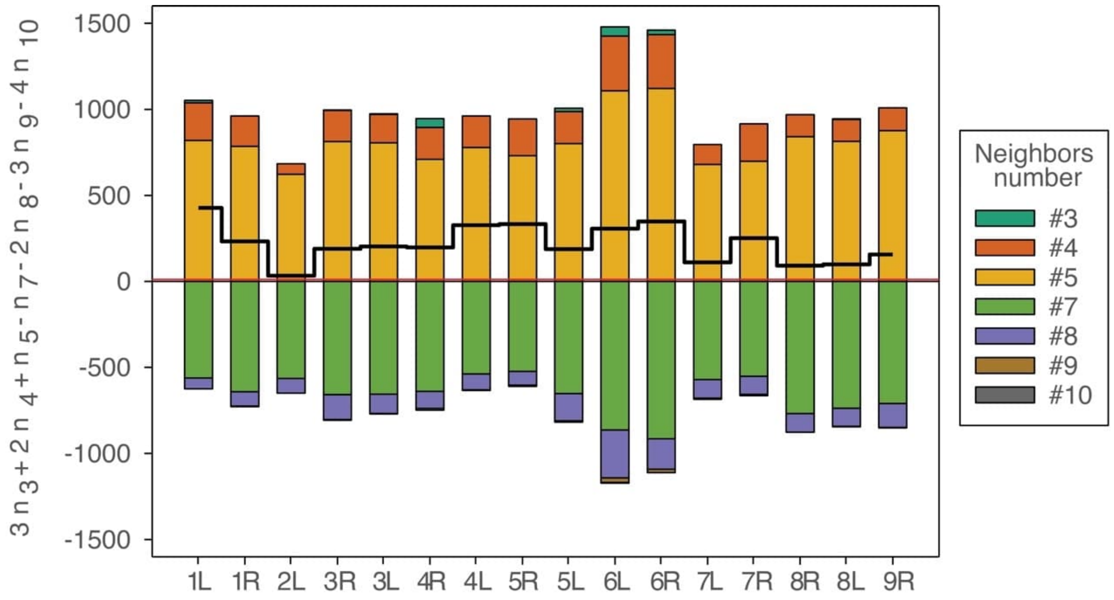

Supplementary Figure S4. Euler sum (black line, calculated from the equation on the y-axis) for all *hyomandibulae* with increasing size from left to right and the contribution to Euler sum by tiles of different edge/neighbor number (indicated by the legend). Geometries that contribute positively or negatively to the Euler sum are shown above or below the horizontal 0 line, respectively. For a closed tiled surface, the Euler sum should equal 12 (thin red line, very close to the 0 line at this scale), indicating slight preponderance of geometries with fewer than six sides. In *hyomandibulae*, pentagons, hexagons and heptagons dominate (see Figure 3); tiles with fewer than 4 and more than 8 edges contribute negligibly. Vertical deviation of the Euler sum black line from the horizontal 0 line indicates a departure from the Euler relationship: the graph shows a strong imbalance towards tiles with smaller edge numbers (i.e. pentagons). A possible explanation could be that a small portion of the *hyomandibula* (where a muscle inserts) is not covered by tesserae (see Figure S1F). The imbalance indicates that this uncovered region would —if the regions were to be covered and if the Euler relation were followed— carry comparatively more tiles with higher edge numbers, which makes sense based on its predominantly negative Gaussian curvature (i.e. its saddle-like structure). The large tesserae that dominate and determine the curvatures of some structural ridges (see Figure S1C,D) would also affect the Euler sum.

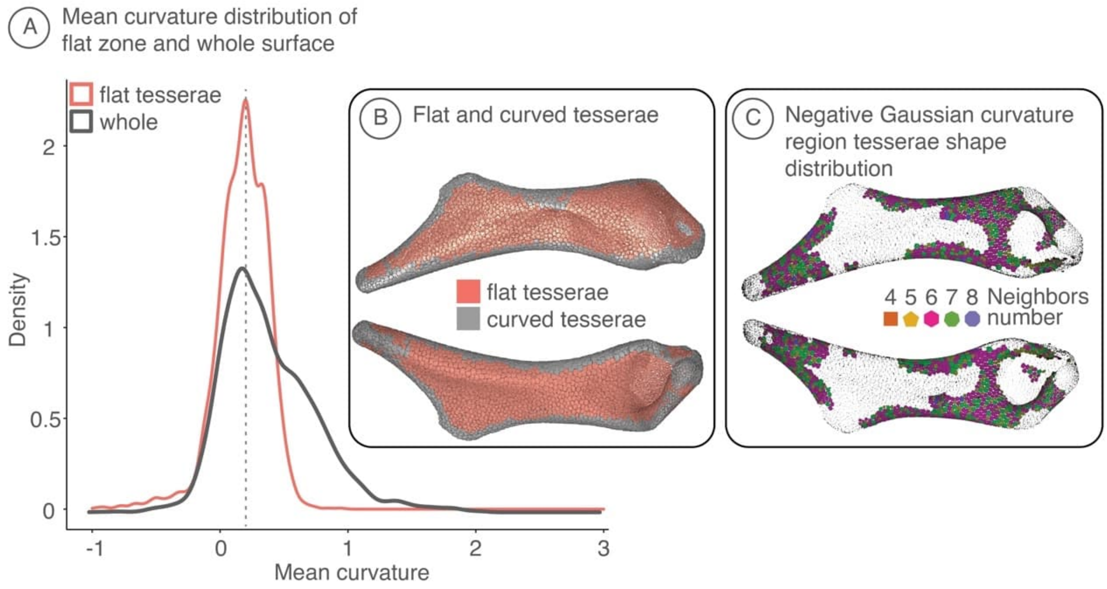

Supplementary Figure S5. Comparison of flat and curved tesserae on an exemplar hyomandibula (Uh#8). A tessera is classified as curved or flat by comparing its width to the local mean curvature value; tesserae are considered curved if this value exceeds sin(10°); otherwise, they are considered flat. (A) The distributions of mean curvatures for all tesserae (dark grey line) vs. only flat tesserae (red line) and (B) a rendering of the hyomandibula coloring regions with curved vs. flat tesserae, show that flat tesserae tend to be localized to near-zero curvature regions. Panel (B) highlights that tesserae identified as curved by this criterion are predominantly located in the large ridge zones of the *hyomandibula*, overlapping with areas of negative Gaussian curvature, as highlighted in panel (C). Note also that in S5C, regions with negative Gaussian curvature consist of pentagons, hexagons and heptagons.

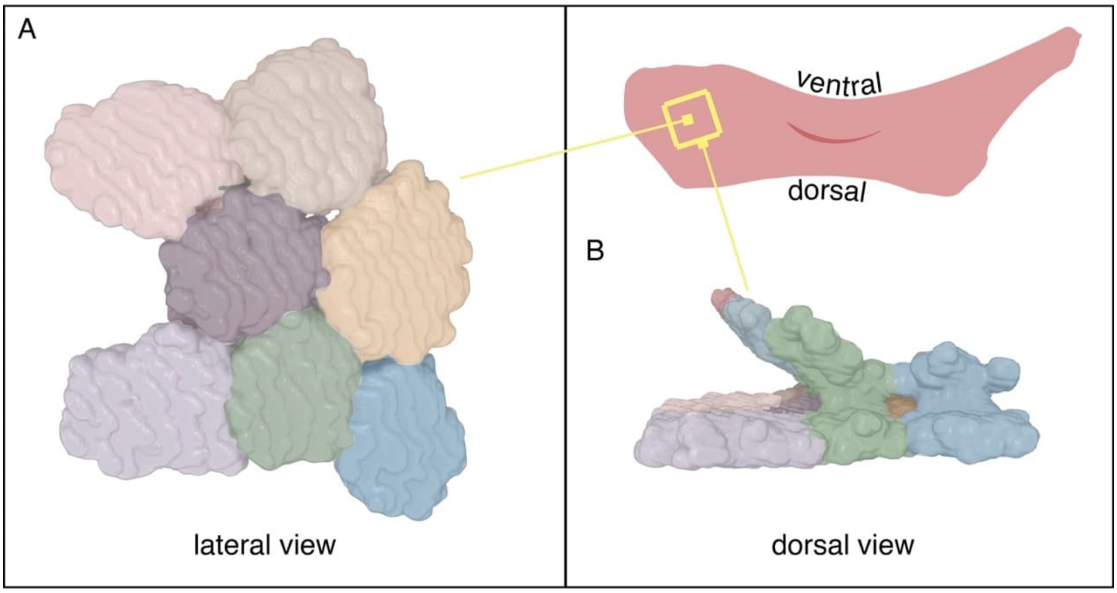

Supplementary Figure S6. Hourglass tesserae, an abnormal morphology observed occasionally at the edge of the medial fossa, connecting tesseral layers on the lateral and medial sides of the hyomandibula. A) A group of tesserae in lateral view (from the *hyomandibula* region shown schematically in the upper right), B) the same tesserae from dorsal view. Note that the three tesserae marked as purple, green and blue at the bottom in A) are the same shown in B); the green and blue tesserae show the hourglass morphology, whereas the purple is a more common plate-like tessera. Tesserae images are volume-rendered from µCT data.

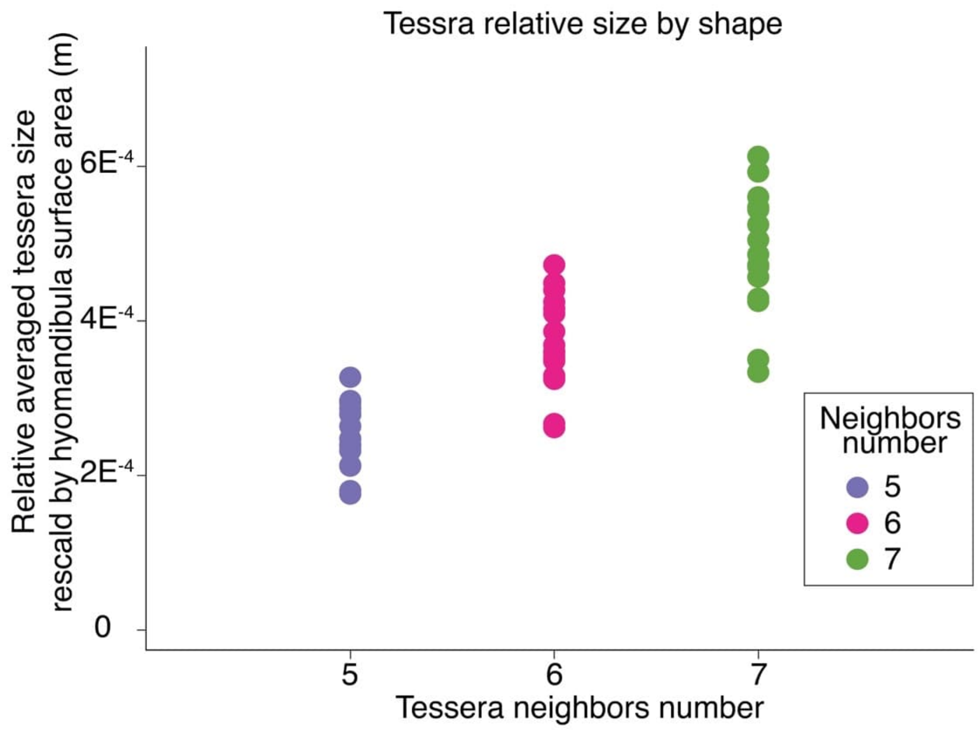

Supplementary Figure S7. The relative average tesserae size increases with the number of neighboring edges, indicating that, regardless of animal age, tesserae with more neighbors tend to be larger.

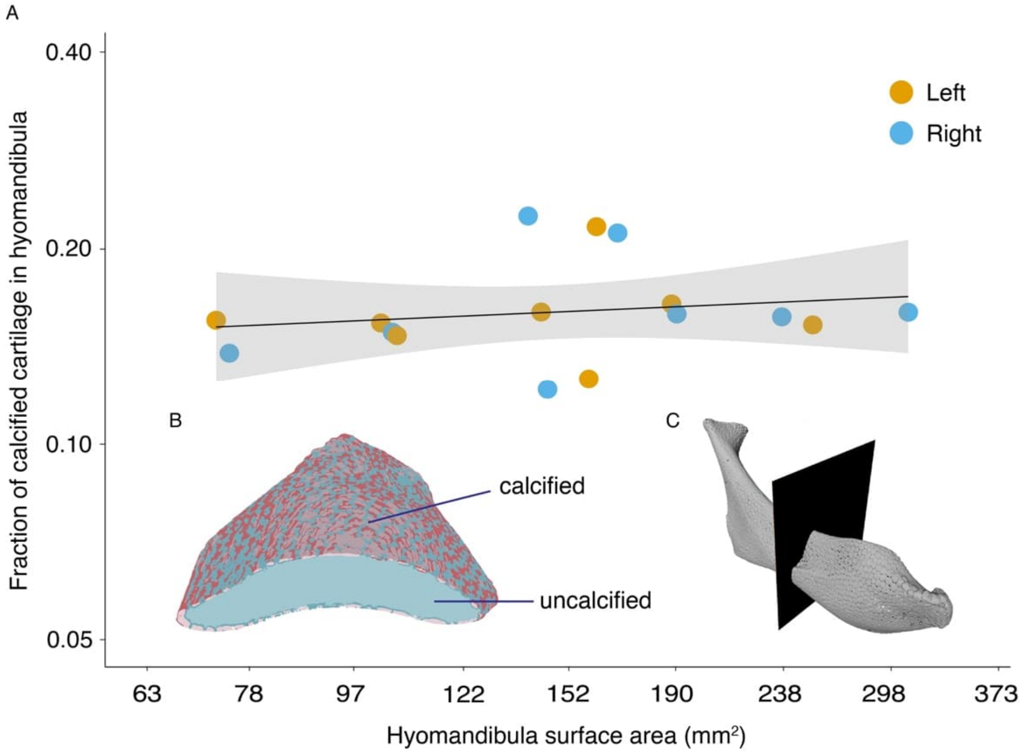

Supplementary Figure S8 Fraction of calcified cartilage (tesserae) to whole *hyomandibula* volume (including tesserae and uncalcified cartilage): the proportion of calcified cartilage in the hyomandibula is relatively consistent across age (median = 0.167 or ~17% of skeletal volume). Both horizontal and vertical axes are logarithmically scaled. (B) cross-section of hyomandibula (C) with calcified cartilage (coral red) and uncalcified cartilage (blue).

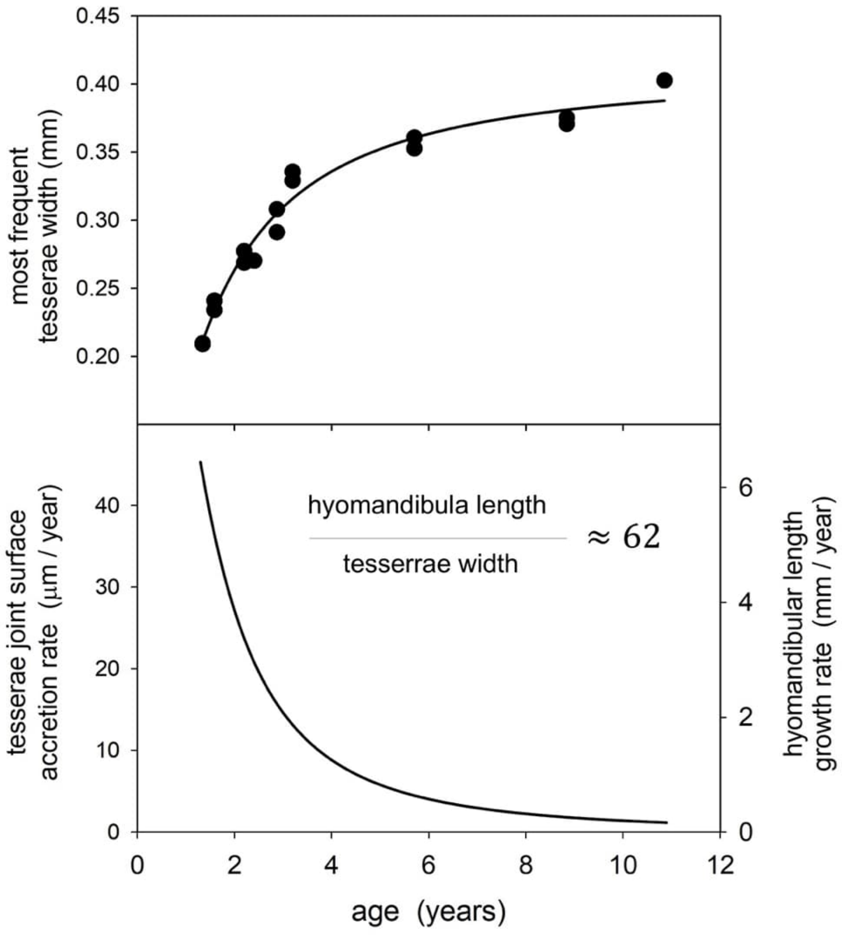

Supplementary Figure S9: A rough estimate for the accretion rate at the tesserae surface, considering a hexagonal tiling with hexagons of side length a and, thus, tesserae width $l = 2a$. The upper graph shows l as a function of animal age from our CT data, empirically fitted by a logistic function with three parameters. The lower graph shows the derivative of this fit function dl/dt (where t is the animal age) multiplied by $\sqrt{3}/4$ to give the mean accretion rate on the joint surfaces of typical hexagonal tesserae (left axis). Since the ratio of hyomandibula length to tesserae width is constant throughout growth (value given in the figure), the same curve also describes the growth rate of hyomandibula length (right axis).

Supplementary TABLE S1. Comparison of the geometric regularity of generalized polygons with tesserae, using a ratio of polygon/tessera area to the longest diagonal (width, in our tesserae measurements; Figs. 4, 6). Ratios for equilateral versions of the most common polygons are listed; these ratios are, on average (0.8465), higher than that calculated from median tesserae dimensions (0.7796), indicating that tesserae are not equilateral (i.e. their shapes are somewhat distorted).

	Equilateral polygon			Tesserae in our dataset
	Five-sided	Six-sided	Seven-sided	$\frac{\sqrt{median\ tesserae\ area\ size}}{median\ tesserae\ width}$
$\frac{\sqrt{A}}{D} = \frac{\sqrt{polygon\ area\ size}}{longest\ diagonal}$	0.810	0.866	0.848	0.7796
Average occurrence in current dataset (see Fig. 3)	25%	50%	20%	
Averaged $\frac{\sqrt{A}}{D}$	0.8465			0.7796

Supplementary TABLE S2. Key Sources Table

HYO #	SA	HL	Tess #	DW	M /F
1L	72.7	13.03	3025	110	M
1R	74.7	13.10	3081	110	M
2L	102.6	15.26	2526	126	M
3R	105.1	15.31	3177	122	F
3L	106.0	15.44	3124	122	F
4R	139.5	17.82	2934	140	F
4L	143.3	17.95	3061	140	F
5R	145.3	17.92	2758	144	F
5L	158.4	17.94	2728	144	F
6L	160.9	18.93	4176	131	F
6R	168.2	18.90	4178	131	F

7L	188.3	20.07	2695	167	M
7R	190.3	20.12	3488	167	M
8R	237.0	23.98	3488	190	F
8L	252.8	24.00	3481	190	F
9R	308.7	26.65	3431	211	M
